# Screen-Cam Imitation Module for Improving Data Hiding Robustness

**DOI:** 10.3390/s25237226

**Published:** 2025-11-26

**Authors:** Kristina Dzhanashia, Aleksandr Fedosov, Oleg Evsutin

**Affiliations:** Tikhonov Moscow Institute of Electronics and Mathematics, HSE University, 20 Myasnitskaya Street, 101000 Moscow, Russia

**Keywords:** data hiding, image-to-image translation, neural networks, robustness, watermarking

## Abstract

**Highlights:**

**What is the main findings?**

- The module for imitating capturing images from screen (screen-cam attack imitation).

**What is the implication of the main finding?**

- The opportunity to increase robustness of neural-networks-based watermarking solutions.

**Abstract:**

Using an attack-simulation module is a well-recognized approach to improving the robustness of end-to-end neural-network-based data-hiding schemes. However, most proposed attack simulators are limited in the types of attacks they cover, usually handling only a basic set of digital transformations. Real, in-demand use cases for data-hiding methods may involve modifications that cannot be modeled by basic digital transformations such as filtering, noise, or compression. In the screen-cam scenario, when an image containing hidden data is displayed on a screen and captured by a camera, the distortions are much more complex and typically require manual experiments that manipulate physical objects in order to replicate. This hinders both the process of creating applicable data-hiding schemes for this scenario and evaluating their effectiveness. In this work, we propose a generator neural network to simulate screen-cam distortions that can replace the manual, time-consuming operations of replicating this attack in the real world, and we show how it can be used to improve the robustness of an existing data-hiding scheme. In our example, we increased robustness by 15% in terms of bit error rate.

## 1. Introduction

In today’s world, users constantly exchange large volumes of multimedia content, leading to cybersecurity threats such as content misuse and intellectual property rights (IPR) appropriation. One approach to combating these threats is the use of digital watermarks. Digital watermarks are information about content or its owners that is added to the content to form a single, inseparable digital object with the content, without changing the significant functional properties of the object itself or hindering its use in authorized scenarios.

The idea of using watermarks to authenticate data is not new; watermarking was recorded as early as 1292 AD [1]. With the advent of digital data exchange, the field of digital watermarking has rapidly developed, with methods being proposed for working with digital images [2,3]. While traditional digital watermarking methods remain relevant for their intended applications, the field itself continues to evolve to address a wider range of scenarios and achieve greater universality of embedding schemes. In recent years, particular attention has been paid to achieving the robustness of watermarks in the case of a watermarked image being captured on a camera and displayed on a screen or a screen-cam attack [4]. This means that there must be a possibility of detecting or extracting the watermark even with such critical changes to the containing content.

On the other hand, the use of neural networks has become one of the dominant paradigms in the field of information embedding. Although the use of neural networks for information hiding is a relatively old research area (e.g., [5]), it has gained particular traction in the last five years due to the ability to work with more computationally demanding neural network architectures and the resulting achievement of outstanding performance indicators.

A typical use case for end-to-end embedding with neural networks involves two networks: an embedding network and an extracting network. These networks are often trained jointly, and the training process involves synchronizing them with each other. In reality, although an embedding and extracting network is sufficient to build a working scheme with high embedding imperceptibility, achieving robustness requires additional measures.

Using an attack-simulation module is a well-established approach to improving the robustness of end-to-end neural-network-based data-hiding schemes. It models possible distortions during training. It receives images with embedded information, applies distortions, and passes them to the extracting network. This approach allows the networks to adapt to the need for robust embedding and, unlike the use of pre-distorted data, does not restrict the embedding process from evolving during training.

However, most existing simulation modules are limited in the range of attacks they produce, typically covering only a basic set of digital transformations. In real-world applications, data-hiding methods often face modifications that cannot be represented by simple digital transformations such as filtering, noise, or compression.

In the screen-cam scenario, where an image containing hidden data is displayed on a screen and captured by a camera, distortions are far more complex and usually require manual experiments involving physical setup adjustments to reproduce. This complicates both the development of practical data-hiding schemes for this setting and the evaluation of their performance.

In this work, we propose a generator neural network that simulates screen-cam distortions, eliminating the need for manual, time-consuming replication of such attacks in real-world conditions. We also demonstrate how this simulator can be used to improve the robustness of an existing data-hiding scheme, achieving a 15% reduction in bit error rate.

The key contributions of this work are the following:

1. A screen-cam distortion simulation module that is independent of any specific data-hiding method
2. Proposed scenarios for using this module to improve data-hiding robustness.
3. A demonstration of enhanced watermarking robustness through the use of the screen-cam distortion simulation module.

The paper is organized as follows. Section 2 reviews various approaches to achieving robustness in data hiding, with particular attention to attack simulators. Section 3 describes the proposed screen-cam distortion imitation module. Section 4 demonstrates the module’s functionality and shows how it can be used to improve the robustness of an existing data-hiding technique through an illustrative example. Finally, Section 5 presents the discussion, and the paper concludes with a summary of the findings.

## 2. Related Works

In this section, the ways through which various neural-networks-based schemes achieve robustness are discussed.

**Figure 1:**
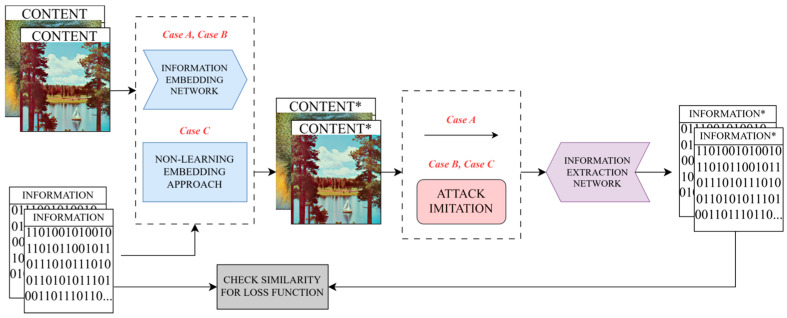
Neural networks-based data hiding. Case A denotes the case without an attack imitation block; Case B with an attack imitation block; Case C corresponds to a case when neural network is not used for embedding. «*» denotes the slight differences between embedded and extracted information, and initial content and content after embedding.

The typical end-to-end neural-networks-based solution includes two main parts: the embedding network, often referred to as an encoder, and the extracting network, also known as a decoder. For this paper, the details of network architectures are mostly irrelevant. The general framework is presented in Figure 1, case A.

The most common approach to achieving robustness in that scenario is the introduction of an attack simulator or attack imitation block, as portrayed in Figure 1, case B.

The following works [6,7,8,9] use the attack imitation block, consisting of standard laboratory modifications.

In [6], a learnable end-to-end model for image steganography and watermarking is proposed. In addition to that shown in Figure 1, it also includes an adversarial discriminator for steganographic purposes. To simulate distortions, a noise layer is applied, consisting of dropout, cropout, crop, Gaussian blur, and JPEG compression. The robustness is demonstrated on 128 × 128 × 3 images with 30-bit messages. It is shown that the model achieves robustness to the distortions it was trained on.

In [7], a framework with residual diffusion watermarking is proposed. Various attack imitation blocks are considered in this work. Three networks are trained with different attacks: the first with Gaussian noise, the second with JPEG compression, and the third with a combination of multiple attacks applied with equal probabilities, namely Salt and Pepper noise, Gaussian noise, JPEG compression, and mean smoothing filter. The resulting networks’ robustness is tested against laboratory attacks; however, the screen-cam scenario is not covered.

In [8], a learning framework for blind and robust watermarking using reinforcement learning is proposed. The framework consists of three stages: watermark embedding, attack simulation, and weight updating. The following attacks are used during training in particular: Gaussian filtering, median filtering, JPEG compression, cropping, rotation, and rescaling. As a result, the proposed approach demonstrated superior robustness compared to analogous frequency-domain-based techniques. Testing was conducted using 24-bit 512 × 512 color images as covers.

The watermarking approach proposed in [9] utilizes pre-processing networks, a watermark embedding network, attack simulation, and a watermark extraction module. The attack simulation includes a group of pixel-value change attacks and geometric attacks. The first group includes Gaussian, average, and median filtering; Salt and Pepper and Gaussian noise; sharpening and JPEG. The second group includes rotation, crop, and dropout with various strengths. The method’s robustness is tested against a set of common laboratory attacks with watermarks having the size of 8 × 8 binary images.

Attack simulation modules that attempt to cover not only laboratory but also screen-cam attack are used in [10,11,12,13,14].

In [10], a watermarking framework with chaotic encryption SE-NDEND is proposed. It employs a noise layer which subjects images to random attacks before they arrive at the decoder. In addition to using common noise attacks, the scheme also contains uniquely designed Moire distortions. The process of simulating Moire distortions includes the addition of line patterns, noise reduction, enhancement, and binarization. The resulting scheme shows robustness against common processing attacks and simulated Moire distortions. The experiments are carried out using 128 × 128 color cover images and 32 × 32 color watermarks.

In [11] a neural-networks-based data hiding scheme for embedding invisible hyperlinks is proposed. It primarily works with embedding 56-bit hyperlinks after error correction and 400 × 400 pixels images. As an attack imitation block, it uses a set of digital transformations that are chosen to simulate screen-cam distortions. It consists of perspective changes, motion/defocus blur, color manipulation, noise, and JPEG compression.

The similar approach to attack simulation as in [11] is also used in [12,13].

In [14], a watermarking framework FPSMark that is designed to withstand the partial screen-cam attack is proposed. It performs a block-wise encoder–decoder training with the noise layer in between. Similarly to [11], a set of digital transformations is chosen to simulate the screen-cam process, including Gaussian blur, perspective distortion, a special algorithm to emulate lightning distortions, brightness, contrast, saturation distortions, Gaussian noise, Moire simulation [15], dropout, resizing, and JPEG compression. The testing is conducted using 400 × 400 images and 100-bit long messages. The ability to extract watermarks from partially captured images is shown.

In some works, neural networks are used exclusively on the extraction side; nevertheless, the attack imitation block principles are still applied to achieve robustness. The principle of those works is illustrated in Figure 1 as case C.

One example of that approach is presented in [16], where extraction is performed through block-wise classification of image blocks using a minimalistic network that is trained on examples that were subjected to a random attack from the attack set. The attack set includes scaling, Wiener filtering, additive Gaussian noise, rotating with cropping, JPEG, and JPEG2000. The resulting scheme obtains robustness to this set of attacks and some that were not used during training.

The usage of neural networks exclusively on one side also allows training samples to be collected independently, outside of the training scheme. In [17], the extraction network was additionally trained using images that were sent through social network channels to obtain robustness against them. This approach, however, is limited by the effectiveness of the embedding method used. In the end-to-end approaches, subjecting training samples to real channels on the run does not seem plausible. Moreover, even when it is possible, the manual collection of a training set may be very time-consuming.

It is worth mentioning that there are some alternative approaches to achieving robustness of end-to-end approaches that are not based on attack imitation.

For example, in [18], an alternative solution is proposed. In this work, robustness is achieved through the usage of an invariant layer in the network architecture. The idea is to overproject the most important information from image space into overfull space and deactivate the neural connections of areas of the original image that are not relevant to the message.

In [19], it is proposed to use an invariant domain that has semantic and noise-invariant information regarding the watermark. To achieve this invariant domain, a self-supervised watermarking framework that simultaneously learns watermarking and invariant domain is developed.

While [18,19] demonstrate very promising alternative approaches to achieving robustness, the scope of this study is limited to attack imitation-based works.

Lastly, the following works experiment with the ideas most closely aligned to the idea of the proposed work.

In [20], a network that simulates the screen-cam is proposed. It is designed to model both photometric and radiometric effects of screen capturing. The distortion network, as well as the embedder and recovery networks, are feature-dense blocks with feature maps at different scales in the shape of a U-Net. The embedder also employs a Siamese architecture. For training the distortion network, 1,000,000 image pairs were used with a loss function that combines similarity between simulated distortions and actually captured images with a quality loss term. The quality loss is calculated by passing images through a pretrained object recognition network and minimizing the differences between feature maps at several levels. The effectiveness of the proposed data hiding solution in the screen-cam scenario under different angles is demonstrated.

In a more recent work [21], the screen-cam attack is simulated with an image degradation module. In this work, the screen-cam is modeled as a combination of image degradation and additive noise with corresponding parameters. The image degradation is performed through a conditional neural network. In the experiments, the proposed noise layer is incorporated into a watermarking framework. The proposed approach differs from ours in the network architecture. It begins with four blocks of joint convolutions and ReLU activations, which then split into two branches: one branch consisting of convolution, ReLU, pooling, and fully connected layers, and the other consisting of two convolution layers with ReLU and reshaping. The outputs of the two branches are then concatenated. The first part serves as a distortion parameter resolver. The second part uses the obtained parameters together with clean images to generate a distorted image using up-sampling and convolutions with ReLU. As a loss function, the mean squared error (MSE) between a clean image and an image subjected to screen-cam distortions is used.

In both [20,21], the loss functions used to train the simulation module are based on measuring the distance between the generated and reference images. However, in real practice there is no single reference image. Depending on shooting conditions such as equipment, lighting, distance, and so on, applying the screen-cam attack to the same image can yield completely different results. Therefore, we believe it is more appropriate to base the loss function not so much on how closely the generated image matches the reference, but on how distinguishable the generated image is from other images obtained after screen capturing. This idea will be developed further in the current work.

As seen from the review, end-to-end neural-network–based solutions that use attack imitation modules are widespread; however, they generally rely on similar digital transformations that do not correspond well to real-world scenarios such as a screen-cam attack. In a few works, techniques for simulating screen-cam distortions have been demonstrated. Some are based on digital transformations and algorithmic solutions, such as [11,14], while others use neural networks, such as [20,21]. Nevertheless, all of them are structurally different from the approach proposed in this work.

In the proposed work, we use neural networks for the purpose of distortions modeling [22]. We apply the conditional generative adversarial network framework pix2pixHD for the training, and the resulting generator for simulations consists of two branches, one for minor details and one for bigger ones, which is a new and different approach to the analogues. The proposed approach is described in detail in the next section.

## 3. Materials and Methods

We view the problem of generating realistic screen-cam images from undistorted images as an image-to-image translation task. A conventional approach to solving this problem is to use convolutional neural networks; however, as shown in [23], using only the distance between predicted and ground truth images as the loss function does not always yield satisfactory results, and designing a more specific loss function presents an additional challenge. Therefore, the commonly used pix2pix framework proposed in [23] can be employed to address various image-to-image translation tasks. It is based on conditional generative adversarial networks (GANs), which differ from non-conditional GANs in that both the generator and discriminator use the input data x (images before transformation), whereas in conventional GANs the discriminator relies only on the generator’s output y (generated images). Its major advantage is that it allows the loss function to be learned during the training process. The conditional GAN has the following objective:

(1) $$L_{cGAN}\left(G,\;D\right)=E_{x,y}\left[\log D\left(x,y\right)\right]+E_{x,z}\left[1-\log D\left(x,G(x,z)\right)\right],$$

where G stands for the generator that learns mapping from input image x and random noise z to the resulting y in a way that would fool the discriminator, and D stands for a discriminator that tries to differentiate between x and y, while E denotes taking the average.

However, it was noted that pix2pix may suffer from a lack of detail and realistic textures in high-resolution outputs [24]. To address this issue, the improved pix2pixHD model was proposed in [24]. It differs from the original pix2pix by incorporating a coarse-to-fine generator, a multi-scale discriminator architecture, and a more robust adversarial learning objective function.

**Figure 2:**
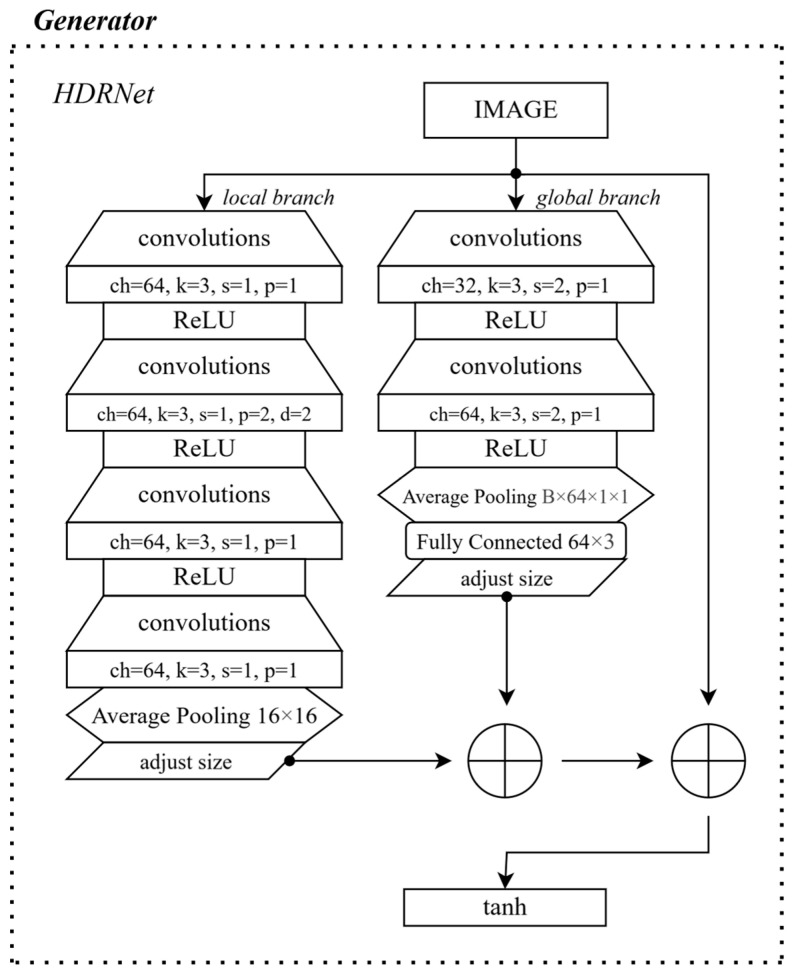
Generator architecture.

In this work, pix2pixHD is used as the foundation for solving the task, with its generator replaced by HDRNet to improve color correction [25]. The main idea is that the input image is first processed through a local branch, after which the resulting activation map is bilinearly interpolated back to the original image dimensions. In parallel, the image passes through a global branch and a fully connected layer to form an activation map that captures more global patterns. The resulting activation maps, which match the original image size, are then combined pixel by pixel with the input image to create color transformations. The generator structure is illustrated in Figure 2.

As a discriminator, three NLayerDiscriminators are used. They analyze images at various scales and downscale them through two-directional average pooling. Each NLayerDiscriminator contains convolutional layers with normalization and LeakyReLU, gradually increasing the number of filters. The final convolutional layer outputs a probability map that defines the image’s “realism”.

The discriminator’s final operation is as follows: the input image and the final training image are concatenated along the channel axis, producing a true pair. A fake pair is created similarly from the input image and the image generated by the model. Each pair is then fed to the discriminator, which produces activation maps and probability distributions for each patch in each pair.

Moreover, the spatial transformer network (STN) was used for perspective correction. It compensates for geometric distortions using trainable affine transformations. The STN was used exclusively in the reverse direction; that is, it was applied to correct perspective distortions during training. Spatial transformations simulating the real-world effect of screen capture were added to the images automatically by generating random transition matrices. This significantly improved the realism of the distortions without the need to train an additional module.

The generator’s loss function included a combination of GAN loss and feature matching loss (2). The former helped to train the generator to produce realistic images capable of fooling the discriminator. The latter compared the internal representations of the ground truth and the generated image pair, further stabilizing the training and improving the detail of the output as follows:

(2) $$L_{G}=L_{GAN}+\lambda_{feat}\times L_{FM}+\lambda_{L1}\times L_{L1}$$

where $L_{GAN}$ is a main GAN loss, $L_{FM}$ is a feature matching loss (the difference between the features extracted by the discriminator from real and generated images), $L_{L1}$ is the mean absolute error between the generated and reference images, and $\lambda_{feat}$ = 10, $\lambda_{L1}$ = 2 are weights.

During preliminary experiments with pix2pix architecture, the resulting images were not of satisfactory quality as the model gave too much attention to the pixel-by-pixel correspondence. Here, the $L_{GAN}$ component helps to view the problem more broadly than pixelwise similarity. It is possible to conduct further experiments with different weight settings of the loss function to study the impact on the resulting images.

**Figure 3:**
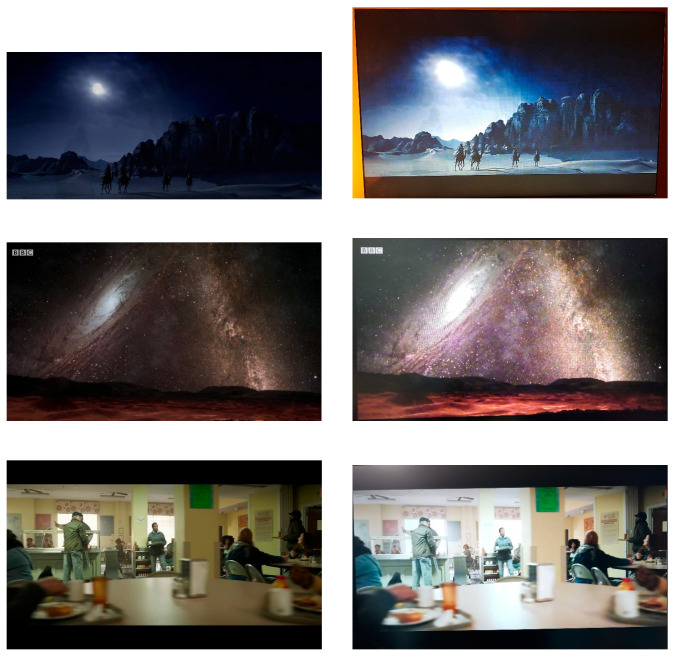
Training dataset pairs of original and captured images.

The model was trained over 200 epochs on a dataset containing realistic screen-cam distortions (Figure 3). A proprietary dataset, comprising 1000 pairs of FullHD images where the original digital images and their distorted versions, captured by capturing a monitor screen with a mobile phone camera, served as the basis. Seven monitors and three mobile devices were used to assemble the dataset. The dataset was then expanded through augmentation.

The Adam optimization algorithm was used with the initial parameters: learning rate $lr=10^{-4}$ and coefficients β=(0.5, 0.999). The batch size was 4 images. During training, the generator adapted to create images that were as close as possible in visual characteristics to screen shots, preserving the scene structure, illumination, and color saturation.

The details of training are as follows. The architecture of networks and their depth are as described previously.

Optimizer: Adam optimizer.

Learning rate: $10^{-4}$.

Coefficients: β=(0.5, 0.999).

Batch size: 4.

Training was conducted using Python 3.9 in Jupyter Notebook 6. The local environment was a MacBook Pro with an Apple M1 processor (8-core CPU, 8-core GPU, 16-core Neural Engine) running macOS (Apple Inc., Cupertino, CA, USA). Computations were performed on Google Colab (free tier, Mountain View, CA, USA), which provides dynamically allocated CPU/GPU resources (about 12 GB RAM, exact GPU type varies). The following key frameworks and libraries were used with these versions to run the model: PyTorch 2.8.0, TorchVision 0.23.0, NumPy 2.0.2, OpenCV 4.12.0.88, Pillow 11.3.0, NetworkX 3.2.1, SymPy 1.14.0, Filelock 3.19.1, fsspec 2025.10.0, Jinja2 3.1.6, MarkupSafe 3.0.3, mpmath 1.3.0, and Typing Extensions 4.15.0.

**Figure 4:**
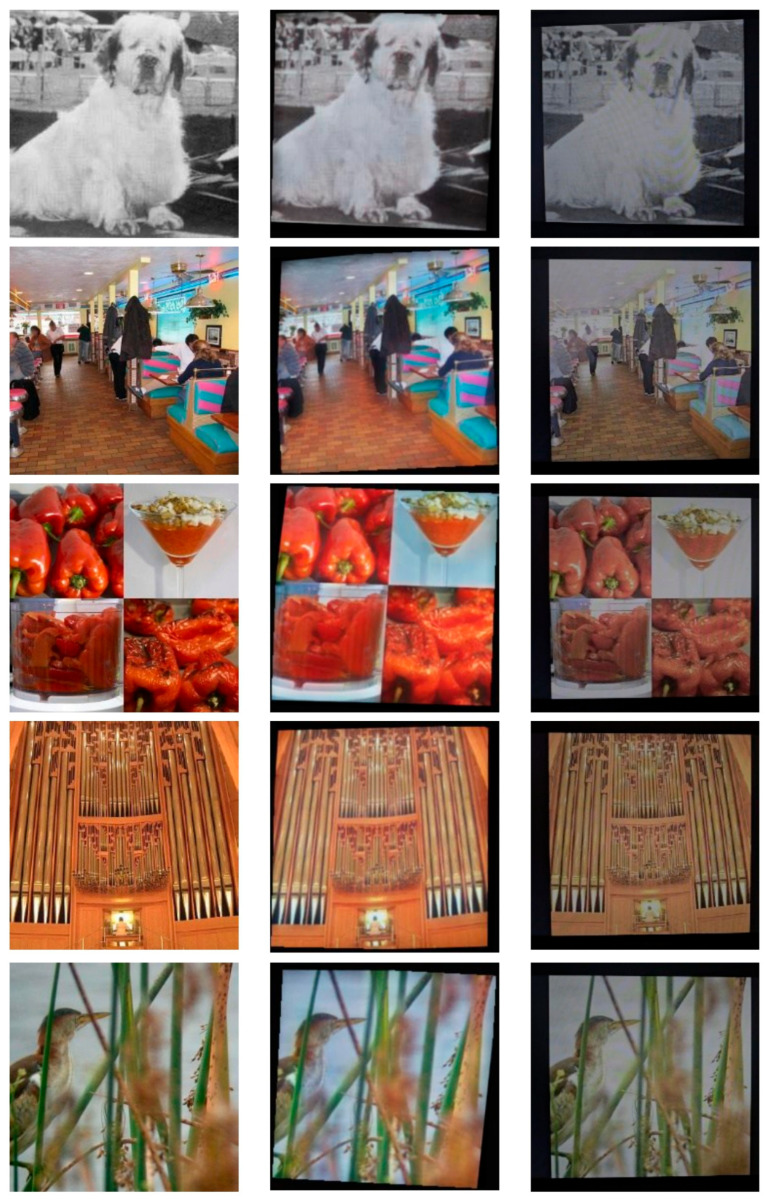
Output examples: in the first column are initial images, in the second column are the module’s outputs, and in the third column are the results of a real screen-cam attack.

Examples of the module’s output, the original (undistorted) images, and the images obtained from an actual screen-cam attack are shown in Figure 4. Note that the example images were not used during training, and the results of the actual attacks on those images were not used to produce the module’s outputs. This explains the differences in shooting conditions between the real captures and the simulated results.

In the next section, the application of the proposed module is shown.

## 4. Results

Further, we present experiments that demonstrate the effectiveness of the developed screen-cam imitation module in improving the robustness of a data-hiding scheme against screen-cam distortions.

First, we describe the neural-network-based end-to-end watermarking method chosen for the experiments. More details on the implementation of the watermarking method can be found in [26]. This method processes images block by block, embedding two watermark bits into each 32 × 32 image block. The bits are first represented as a two-channel binary image of the same size as the block. The encoder network consists of 35 layers, including consecutive convolutional blocks, batch normalization, and clipped ReLU layers. Additionally, weighted addition layers are employed so that the coefficients for merging the watermark-bit and cover-image branches are learned during training. As a result, a modified image block containing the two watermark bits is produced. Since the embedding process is not the main focus of this paper, it is not discussed further.

The decoder is a convolutional neural network that functions as a multi-label classifier, assigning two extracted bits to each image block. Its architecture is shown in Table 1. The network outputs two values between 0 and 1, which are then rounded to obtain the extracted bits. It contains 282.4k parameters and 23 layers, making its training relatively computationally efficient.

During the preliminary joint training of the embedding and extraction networks, a conventional attack imitation block was used. This block applied JPEG compression with a randomly selected quality factor between 80 and 100, together with one additional random attack from the list presented in Table 2.

**Figure 5:**
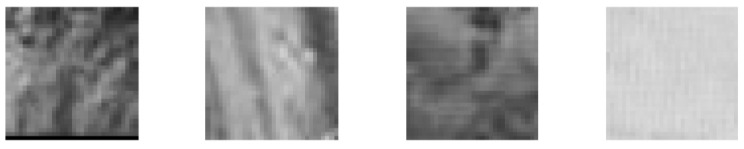
Examples of training blocks.

**Figure 6:**
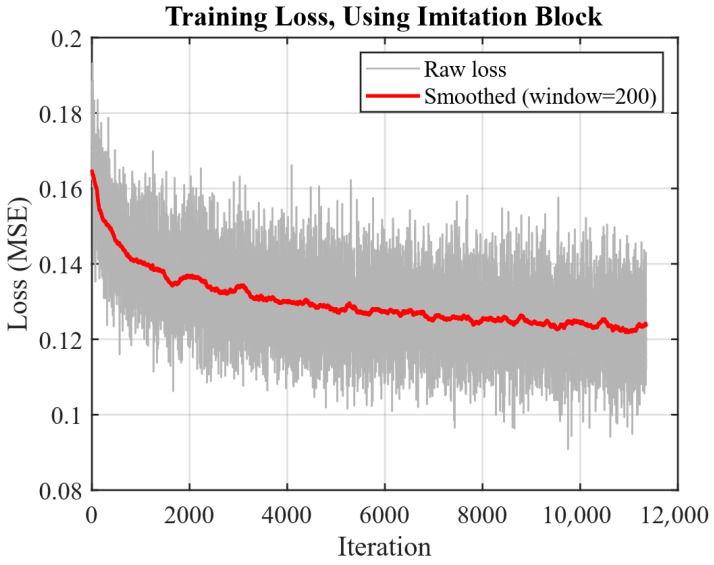
Training loss.

To test the effectiveness of the proposed approach, we perform additional training of the pre-trained extracting network. We embed random 512-bit sequences in 512 × 512 color images that were obtained through cropping and scaling from ImageNet [27]. We use around 800 such images and pass them through the screen-cam imitation module with perspective correction afterwards. The extraction network trains on blocks, so the images are divided into image blocks. For every possible two-bit combination (00, 01, 10, 11) 50,000 blocks are picked for training with 200,000 blocks used overall, the examples are given in Figure 5. During additional training the Adam optimizer with learning rate = 0.001 and batch size of 256 are used. The MSE between the real bits and the network’s outputs before rounding is used as a loss function. Figure 6 shows the progression of the loss function during training. Each iteration corresponds to processing one data batch, computing the loss, and updating the network’s weights.

The implementation of the simulation model in the proposed scenario is as follows:

1. Perform watermark embedding in k 512 × 512 images $I=\left\{I_{1},\;I_{2},\ldots I_{k}\right\}$ as per the watermarking algorithm [26], obtaining $I^{\prime }=\left\{{I^{\prime }}_{1},\;{I^{\prime }}_{2},\ldots {I^{\prime }}_{k}\right\}$. We set k=800.
2. Pass $I^{\prime }=\left\{{I^{\prime }}_{1},\;{I^{\prime }}_{2},\ldots {I^{\prime }}_{k}\right\}$ to the trained generator network, obtaining the result of screen-cam simulation $I^{sc}=\left\{I_{1}^{sc},\;I_{2}^{sc},\ldots I_{k}^{sc}\right\}$.
3. As the extraction network in the considered watermarking approach uses blocks, divide each image from $I^{sc}=\left\{I_{1}^{sc},\;I_{2}^{sc},\ldots I_{k}^{sc}\right\}$ into 32 × 32 blocks, obtaining $b=\left\{b_{1},\;b_{2},\ldots b_{256k}\right\}$.
4. For each possible extraction network output (00, 01, 10, or 11) take 50,000 blocks out of b, obtaining $b_{00}$, $b_{01}$, $b_{10}$, $b_{11}$.
5. Take the pre-trained extraction network and perform additional training on it using $b_{00}$, $b_{01}$, $b_{10}$, $b_{11}$ as a training set with Adam optimizer, learning rate = 0.001 and batch size of 256.
6. Use the obtained extraction network instead of the original one when extracting watermarks.

We compare the extraction networks performances before and after additional training. To evaluate robustness, the bit error rate (*BER*) metric is used. It is calculated as follows:

(3) $$BER=\frac{the\;number\;of\;wrongly\;extracted\;bits}{the\;total\;number\;of\;embedded\;bits}.$$

**Figure 7:**
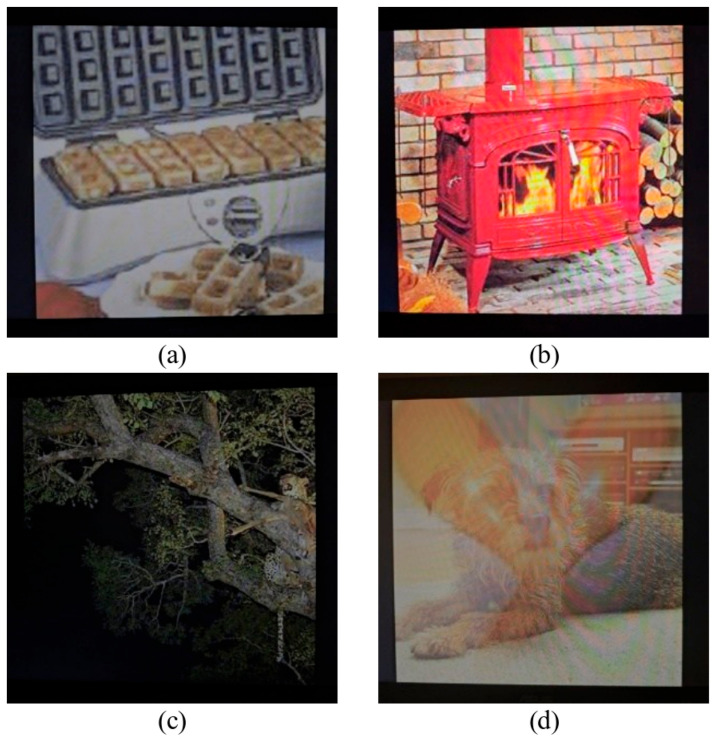
Examples of watermarked images that were shown on a screen and captured on a mobile device. The capture is performed at a distance of 30 ± 5 cm with minimal angle in a dark room. Images (**a**,**b**) are examples of images for which extraction works successfully with lower BER. Images (**c**,**d**) are examples of images for which extraction performs poorly with BER around 50. With image (**c**) geometric synchronization fails because the image merges with the background. With image (**d**) the capturing is done too late and two images overlap.

We pick 1000 actually captured images with a wide range of BER values before training to observe the impact not only on normal cases, when capturing performed successfully, but also on edge cases when the watermark cannot be extracted at all with the initial extracting network. The BER values range from 4.68 to 54.68 with an average of 22.73 and median of 21.67. All images are captured in a dimly lit room, at a distance of 30 ± 5 cm with a minimal angle using a Samsung Galaxy S23 Ultra camera and an Asus IPS HDMI 23″ monitor. The examples of these cases are shown in Figure 7. Prior to the extraction, an automatic perspective correction is performed using speeded up robust features (SURF) [28] according to Algorithm 1.
**Algorithm 1.** Perspective correction with SURF.       Input: $Image_{c}$ is the watermarked distorted image, $Image_{o}$ is a reference image before watermarking.       Output: $FixedImage_{c}$.       Step 1. Set parameters ranges: $t_{values}=[1800,1500,1000]$ are thresholds for SURF feature detection, ${maxRatio}_{values}=[1,0.9,0.8]$ are ratio thresholds for feature matching, ${matchThreshold}_{values}=[0.5,1.5,5,10]$ are match acceptance thresholds.       Step 2. Resize $Image_{c}$ to 512×512 pixels to optimize processing speed.       Step 3. Convert $Image_{c}$ and $Image_{o}$ to grayscale.       Step 4. Initialize values for storing best results as BestInliersCount=−1, $FixedImage_{c}=[].$       Step 5. For each t in $t_{values}$:       Step 5.1. $Points_{c}\leftarrow detectSURFfeatures(Image_{c})$       Step 5.2. $Points_{o}\leftarrow detectSURFfeatures(Image_{o})$       Step 5.3. If number of $Points_{o}<3$ or number of $Points_{c}<3$, return to Step 5.       Step 5.4. $\left[features_{c},validPoints_{c}]\leftarrow extractFeatures(Image_{c},Points_{c}\right)$       Step 5.5. $\left[features_{o},validPoints_{o}]\leftarrow extractFeatures(Image_{o},Points_{o}\right)$       Step 5.6. For each maxRatio in ${maxRatio}_{values}$:       Step 5.6.1. For each matchThreshold in ${matchThreshold}_{values}$:       Step 5.6.1.1. $indexPairs\;\leftarrow \;matchFeatures(features_{c},\;features_{o},\;\;matchThreshold,\;maxRatio)$       Step 5.6.1.2. If number of indexPairs<3 return to step 5.6.1.       Step 5.6.1.3. $transform,\;inliers\leftarrow estimateGeometricTransform(validPoints_{c},\;validPoints_{o})$       Step 5.6.1.4. if number of inliers>bestInliersCount       Step 5.6.1.4.1. $FixedImage_{c}\leftarrow imwarp(Image_{c},\;transform)$       Step 5.6.1.4.2. $t_{best}\leftarrow t$       Step 5.6.1.4.3. ${maxRatio}_{best}\leftarrow maxRatio$       Step 5.6.1.4.4. ${matchThreshold}_{best}\leftarrow matchThreshold$       Step 5.6.1.4.5. bestInliersCount←numberofinliers       Where for imwarp, estimateGeometricTransform, extractFeatures, and detectSURFfeatures their Matlab R2022b (Natick, MA, USA) realization are used.

**Figure 8:**
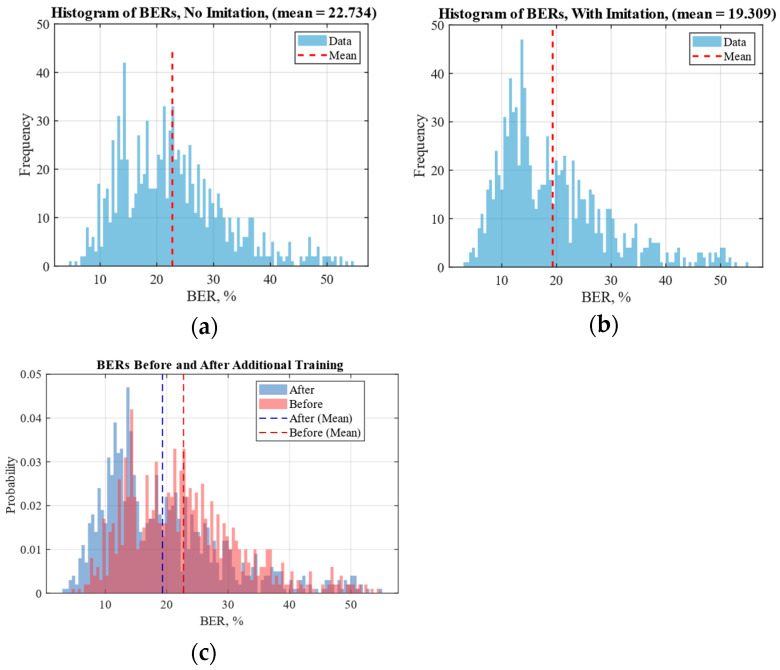
Histograms: (**a**) before additional training with the proposed screen-cam imitation module; (**b**) after the training; (**c**) combined.

The histograms of BERs before and after additional training for 1000 images are shown in Figure 8. As can be seen from the histograms, using the proposed screen-cam imitation module lowered the mean BER by more than 3.4, or by 15%. This demonstrates that the proposed approach can indeed significantly improve robustness to a screen-cam attack.

From the combined histograms, it can be observed that, after training, the peak of the histogram becomes more pronounced and shifts to lower BER values.

Nevertheless, the screen-cam imitation module does not significantly help with edge cases where either the extraction or perspective correction fails in the first place, as the bar levels in the 40–50 BER region remain roughly the same, which is to be expected. We assume that some of the worst-case scenarios cannot be mitigated by the watermarking scheme. For example, if an image is captured in such poor quality that it becomes unusable for any practical application, the presence of a watermark becomes irrelevant, and its loss, reflected by high BER values, is no longer a significant issue. In other cases, where the image remains recognizable, but watermark extraction still fails, potential improvements may lie either in the watermarking algorithm or in the perspective correction system. One way to enhance the latter is by embedding additional orientation marks into the images to assist during the correction process. The alternative approach is to develop a neural network-based solution for geometry alignment. As for watermarking itself, there is a broad research field dedicated to improving robustness, with common techniques including watermark repetition, data scrambling to prevent large consecutive data losses, and the use of error-correcting codes. In the future, it will be possible to add simulation of extreme cases, such as the obscured screen view, to study the limitation of watermarking in such situations.

**Figure 9:**
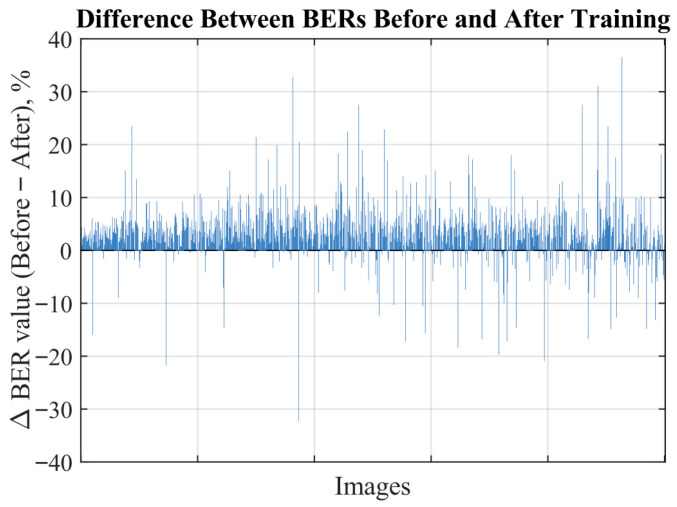
Delta plot.

The delta plot in Figure 9 illustrates the differences in BER values before and after additional training for each individual image. Each bar above zero indicates an increase in robustness, while bars below zero indicate a decrease. It can be observed that most bars are positioned above zero. Although a few individual cases show a decrease, they do not outweigh the overall positive trend.

Table 3 presents the statistical characteristics of the BER values obtained before and after additional training for 1000 images.

The standard deviation remains relatively unchanged and high, which can be attributed to the significant variability typical of the screen-cam channel. Notably, the median BER decreases substantially after additional training with the screen-cam imitation block (by nearly 22%) further emphasizing the benefit of using the proposed imitation module.

To confirm our findings, we performed several statistical tests [29], as shown in Table 4. We refer to the BER values obtained before additional training as b, and those obtained after additional training with the imitation module as a. The tests were carried out on all BER values (1000 samples each for a and b) and also on smaller datasets, where 100 pairs of a and b values for the same images were randomly selected. All tests were implemented using Matlab standard functions. We used a 5% significance level everywhere.

Finally, to confirm that the proposed approach works with other acquisition devices that were not used during training and testing before, we ran tests on four various monitor–mobile phone combinations with 10 random images per each. The results are presented in Table 5.

As a side effect, the additional training with the proposed module allows an increase in robustness to typical laboratory attacks, as shown in Table 6. Although this was not a direct purpose of the module, it does help to increase robustness to laboratory attacks.

To sum up, the results confirm that the proposed screen-cam imitation module effectively enhances robustness against real screen-cam distortions. The next section discusses these findings in greater detail.

## 5. Discussion

The module enhances the robustness of the neural watermarking scheme by 15% through a simple two-step process as follows: running the screen-cam imitation on watermarked images to collect training samples and performing additional training of the extracting network. At the same time, the particularities of the embedding and extracting networks were not essential for running this process. Indeed, the proposed solution can be used with a wide range of end-to-end, neural-network-based data-hiding approaches. The demonstration of the module’s applicability with alternative data embedding schemes can be considered a subject for separate research; nevertheless, in general, whenever a conventional attack-simulation module can be used, our module can also be used as a substitute.

The developed screen-cam imitation module can be applied in data hiding in three main ways:

1. To approximately assess the robustness of a data-hiding scheme to screen-cam. The use of the screen-cam imitation module can help optimize the time and effort spent on manual screen-cam robustness checks. Although, at this stage of development, the module cannot replace full-scale experiments under different angles and distances, it can provide a preliminary evaluation of a scheme’s functionality in screen-shooting scenarios.
2. To train and/or optimize the extraction process. Using an existing data-hiding scheme, the extraction process can be optimized and further trained with the imitation module. Some end-to-end approaches even allow separating the extraction network after the main training process and retraining it with samples obtained from the emulation module, as shown in the previous section.
3. To be used as or together with an attack imitation module inside the main training process of an end-to-end, neural-network–based solution. In the first two cases, the screen-cam imitation module could be replaced with time-consuming manual operations. In this case, however, capturing images from a screen that contains continuously changing embedded messages during the training process is hardly feasible. Thus, this appears to be the scenario in which the proposed module could serve best.

In the previous section, only the second application was covered. In the future, we plan to explore the other two use cases in more detail. The module can also be used beyond watermarking, for example, for solving tasks related to pattern recognition in noisy or distorted images.

Moreover, the neural architecture used to create the screen-cam imitation module can be reused to learn distortions typical of other communication channels in addition to screen-cam. This is an advantage of the proposed approach compared to selecting a set of algorithms to simulate the screen-cam process, as in [11] or [14]. We plan to collect training samples for another commonly addressed attack, print-cam, and retrain the same network to handle it in the near future.

The screen-cam imitation module could be further improved by expanding the training dataset to include a wider range of acquisition devices and lighting conditions, thereby enhancing the model’s generalization and practical applicability.

**Figure 10:**
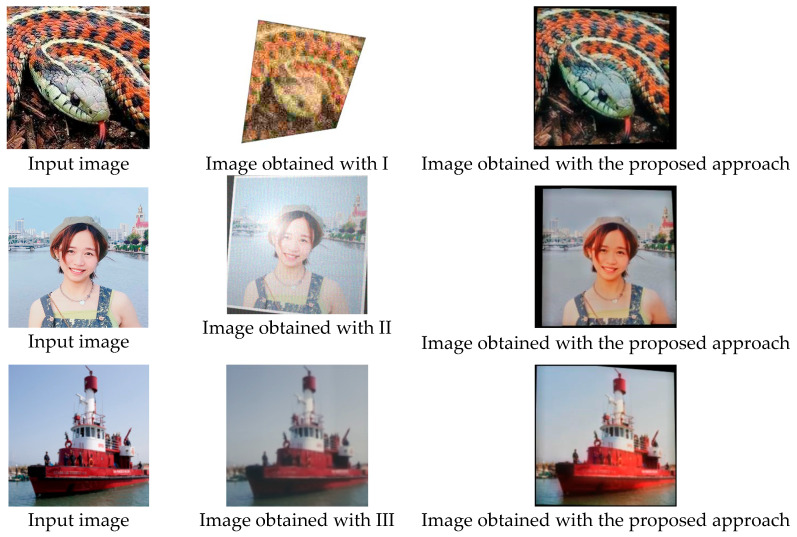
Examples of images generated through alternative approaches (I [11], II [14,15], III [21]) and the approach proposed in this work.

The main differences between the proposed approach and analogues are summarized in Table 7. The visual differences are presented in Figure 10.

## 6. Conclusions

The screen-cam imitation module based on the pix2pixHD image-to-image translation framework with HDRNet is proposed. It can generate images that closely resemble those actually captured from a screen.

We show that additional training with module-generated samples enhances the robustness of the existing data-hiding solution. The mean BER decreased by 15% (from 22.7% to 19.3%) in the presence of a screen-cam attack. The difference between BERs before and after additional training was statistically significant, confirmed using the Wilcoxon signed rank test, Wilcoxon rank sum test, and paired-sample *t*-test at a 5% significance level. In all cases, p-value was below $10^{-3}$.

Two alternative applications are also proposed: improving robustness by integrating the module into the main training process or using it to approximate the evaluation of an existing data-hiding approach’s robustness to screen-cam distortions. In future work, we plan to retrain the module to imitate print-cam and print-scan distortions as well, using a new database.

## Figures and Tables

**Table 1:** The extraction network architecture.

No.	Layer	Output (Height × Width × Channels)	Parameters (No Padding)
0	Input Layer	32×32×1	-
1	Convolution–Batch Normalization–ReLU	28×28×128	1285×5×1 convolutions, stride [1 1]
2	Convolution–Batch Normalization–ReLU	24×24×64	645×5×128 convolutions, stride [1 1]
3	Convolution–Batch Normalization–ReLU	20×20×32	325×5×64 convolutions, stride [1 1]
4	Convolution–Batch Normalization–ReLU	16×16×16	165×5×32 convolutions, stride [1 1]
5	Convolution–Batch Normalization–ReLU	12×12×16	165×5×16 convolutions, stride [1 1]
6	Convolution–Batch Normalization	8×8×8	85×5×16 convolutions, stride [1 1]
7	Convolution–Batch Normalization	8×8×2	21×1×8 convolutions, stride [1 1]
8	Average Pooling	4×4×2	stride [2 2]
9	Fully Connected Layer	2×1	-
10	Sigmoid	2×1	-

**Table 2:** Attacks in the attack imitation block.

Attack	Randomly Changing Attack Parameter	Minimum Value of the Parameter	Maximum Value of the Parameter
None	-	-	-
JPEG2000	Compression ratio	4	16
Scaling with returning	Scaling factor	0.2	0.9
Rotation	Angle	0	2
Winner Filter	-	-	-
Gaussian noise	Dispersion	0	0.01
“Salt and Pepper” noise	Strength	0.02	0.1

**Table 3:** Statistics, BER values (%).

	Mean	Median	Standard Deviation
Before additional training	22.7344	21.6797	9.1686
After additional training	19.3086	16.9922	9.8363

**Table 4:** Statistical tests.

Test	The Null Hypothesis	Samples	p-Value	Conclusion
Two-sided Wilcoxon rank sum test (Mann–Whitney U-test)	Data in a and b are samples from continuous distributions with equal medians	1000 100	$2.8\cdot 10^{-22}$ $4.6\cdot 10^{-4}$	The null hypothesis is rejected
Two-sided Wilcoxon signed rank test	Data a−b comes from a distribution with zero median	1000 100	$6.5\cdot 10^{-91}$ $6.8\cdot 10^{-10}$	The null hypothesis is rejected
Paired-sample t-test	Data in a−b comes from a normal distribution with mean equal to zero and unknown variance	1000 100	$6.6\cdot 10^{-72}$ $6.6\cdot 10^{-7}$	The null hypothesis is rejected

**Table 5:** Tests with various acquisition devices.

Experiment Setting	Mean BER Before Additional Training, %	Mean BER After Additional Training, %
Images shown on HP Pavilion Aero Laptop 13″ and captured on Galaxy A30s	14.14	11.09
Images shown on 32″ LG 32LM6380PLC and captured on Galaxy A30s	22.35	19.7
Images shown on HP Pavilion Aero Laptop 13″ and captured on Huawei nova Y70	17.54	15.17
Images shown on 32″ LG 32LM6380PLC and captured on Huawei nova Y70	16.75	15.76

**Table 6:** Module influence on robustness to laboratory attacks on 100 images.

Attack	Mean BER Before Additional Training, %	Mean BER After Additional Training, %
JPEG compression with Quality Factor = 50	11.69	9.02
JPEG compression with Quality Factor = 80	9.72	7.59
Gaussian noise with variance = 0.001	11.86	9.41
Gaussian blur 3 × 3	10.21	7.81

**Table 7:** Comparison with analogues.

	Approach	Key Differences with the Proposed Approach
[11]	Digital transformations with variable parameters, including perspective warp, motion/defocus blur, color manipulation, noise, JPEG compression	(+) Computationally easier (−) The imitation abilities are limited by the chosen set of digital transformations (−) To simulate a different type of distortion, a new set of digital transformations must be chosen manually
[14,15]	Digital transformations with variable parameters, including Gaussian blur, geometric distortions, brightness, contrast, saturation distortions, Gaussian noise, Moire distortions, JPEG, dropout, cropping	(+) Computationally easier (−) The imitation abilities are limited by the chosen set of digital transformations (−) To simulate a different type of distortion, a new set of digital transformations must be chosen manually
[20]	Distortion network consisting of feature-dense blocks with feature maps at different scales in the shape of a U-Net	(−) The loss function is based on the proximity of generated and reference images, while in the proposed work the loss function also takes into account the ability to differentiate between real screen-captured images and generated images
[21]	Resolver-simulator framework: the resolver generates a degradation parameter, and the simulator is a conditional network that produces screen-shooting degradation using this parameter	(−) The loss function is based on the proximity of generated and reference images, while in the proposed work the loss function also takes into account the ability to differentiate between real screen-captured images and generated images

## Data Availability

The developed module is publicly available at https://github.com/DzhanashiaKrsitina/screencamimitation (accessed on 18 October 2025).

## Abbreviation

The following abbreviation is used in this manuscript:

BER	Bit error rate
